# Handwashing among schoolchildren in an ethnically diverse population in northern rural Vietnam

**DOI:** 10.3402/gha.v6i0.18869

**Published:** 2013-01-31

**Authors:** Le Thi Thanh Xuan, Luu Ngoc Hoat

**Affiliations:** 1 Department of Environmental Health, Institute for Preventive Medicine and Public Health, Hanoi Medical University, Hanoi, Vietnam; 2 Department of Biostatistics and Medical Informatics, Institute for Preventive Medicine and Public Health, Hanoi Medical University, Hanoi, Vietnam

**Keywords:** handwashing with soap, schoolchildren, rural Vietnam, multiethnic population

## Abstract

**Background:**

Handwashing with soap (HWWS) is a simple and effective measure to prevent transmission of fecal–oral disease and other infectious diseases in school-age children. To promote the behavior, we need to understand their HWWS compliance. The aim of this article is to describe handwashing behavior and HWWS compliance and to identify associated factors among schoolchildren in the multiethnic rural area of northern Vietnam.

**Methods:**

The study was conducted in six primary and secondary schools and in the homes of four ethnic villages in northern Vietnam. Quantitative methods included face-to-face interviews with, and demonstration of handwashing protocol to, 319 schoolchildren in first, fourth, and seventh grades. Qualitative methods included structured observations at six schools and 20 homes comprising 24 children. The dependent variable was the self-reported HWWS behavior (yes/no). The independent variables included grade, school type, gender, ethnicity group, owning home latrine, and household assets. Logistic regression modelling was performed to examine associations between HWWS behavior and demographic factors.

**Results:**

Among the 319 schoolchildren interviewed, 66% reported HWWS. Through the demonstration protocol, only 10 out of 319 schoolchildren, performed HWWS satisfactorily. The percentage of students who washed their hands at recommended times (30–60 sec) was 58%. This proportion increased by grade (from 34% among grade 1 to 67% among grade 7; *p*<0.05). Correlates of self-reported HWWS were more common in higher grades [grade 4 vs. grade 1: odds ratio (OR)=4.14 (2.00–8.56), grade 7 vs. grade 1: OR=7.76 (3.67–16.4)] and less common in ethnic minority groups [Xa Phó vs. Kinh-Tay: OR=0.28 (0.11–0.70)]. All 20 homes of schoolchildren visited had soap and water but none of the six schools had soap for handwashing.

**Conclusions:**

This article describes poor compliance of schoolchildren with HWWS in a multiethnic population in Vietnam. Education on handwashing needs to be prioritized among multiethnic children at school.

Handwashing with soap (HWWS) is important for school-age children in the improvement of health and disease prevention (e.g. diarrhea and gastrointestinal infections), which in turn reduces absenteeism due to illness (1–10). Investing in HWWS is minimal and easy, and HWWS is effective in maintaining health. The practice is significant for schoolchildren, who might suffer a more severe burden of hygiene-related diseases compared to adults (11–13).

Previous studies showed that school-based HWWS promotion had significant effect on HWWS practices of schoolchildren (14) and also that those schoolchildren could act as ‘HWWS messengers’ to their family and community (1, 3, 15–18). However, the HWWS compliance of schoolchildren in the developing countries is still low (19–21). A study conducted in rural Senegal found that out of 3,797 primary pupils observed, only 7% washed their hands with soap after using the toilet. Rates were similar for boys and girls (21). A cross-sectional study among 669 students, grade 1–6, in rural Ethiopia showed that only 15% reported HWWS after defecation (19). A survey in 10 rural primary schools in Cambodia found the potential of public education campaigns, as children in higher grades had a greater tendency to improve on sanitation practices (20). Possible reasons for low rate of HWWS among schoolchildren could be lack of soap and water or absence of norms or culture at school, which could educate the schoolchildren on HWWS. Suggested interventions, thus, should be focusing on the physical environment at school, making water and soap available (22) and inculcating a culture of HWWS at the school level (21).

**Policy recommendations**

The public health designers and promoters should focus on creating school culture in HWWS by:

- Prioritization of the topic in the school curriculum
- Integration of the topic in the school activities, particularly extra-curriculum
- Encouragement of new teaching methods of HWWS to schoolchildren
- Motivation of the behavior at home through role model and peer-to-peer education
- Xa Phó and smaller children should be targeted in school hygiene promotional program
- Also, HWWS behavior measurement needs to be formulated

In Vietnam, HWWS was taught to all first-grade schoolchildren as part of national curriculum through 1-hour sessions, titled ‘Body hygiene’. There were some initiatives focusing on promoting HWWS compliance of schoolchildren in Vietnam (23). However, the HWWS rate among schoolchildren is still low. A survey conducted on hygiene practices at schools in Vietnam in 2007 found that only 12% of students washed their hands with soap after defecation, and the situation was even worse in northern Vietnam (24). Another study in 2008 found that no student washed their hands with soap at school (25). Some factors associated with the poor behavior were lack of availability of, and accessibility to, soap (26) and poor soap management (23). However, due to the large-scale social marketing, handwashing behavior, funded by the World Bank has been thoroughly investigated in the rural Kinh population in Vietnam, but not among the ethnic minority groups (EMGs) (27). This study was therefore carried out to analyze the HWWS compliance of schoolchildren in poor settings where they often suffer from the burden of hygiene-related diseases. This information will help policy makers and public health promoters to design an appropriate and effective hygiene program for the multiethnic population.

## Methodology

### Field sites

The study was carried out in two neighboring rural communities in a northern province of Vietnam that was piloting the national target program of water supply, sanitation, and hygiene. The two communities were chosen because they are worse off than others in the region and also because they are pilot sites for hygiene promotion activities, including the second phase (2006–2010) of school hygiene intervention of the Rural Water Supply and Sanitation National Target Program. The two communities consisted of about 10,000 people living in 39 lowland and highland villages. Eighty percent of the population in the two communities belonged to the EMGs, including the Dáy, Tày, Dao, Xa Phó, H'Mông, and Hoa. The highland villages, where the Xa Phó, H'Mông, and Hoa groups lived, were located farther away from the communal center and had less public services than the lowland villages.

The study was carried out in 6 of 23 schools and 4 of 39 villages in the two communities. Six schools for the study included two secondary schools, two main primary schools, and two satellite primary schools. In each community, the primary school system included one main school located at the center of the community and several smaller branches or satellite schools located in the villages. The selection of schools aimed at diversifying the school type (two secondary and four primary schools, four main schools and two satellite schools), lowland and highland schools (five lowlands and one highland). Table 2 indicates that most children in the six schools belonged to the Tày, Dáy, Xa Phó, and Kinh ethnic groups. The four villages selected for home observation were representatives of the four different ethnic groups (Kinh, Tày, Dáy, and Xa Phó) (Table 1).

**Table 1:** Methods applied in the study

					Methods and sample size			

No.	School type	School area	Ethnic group of student	No. of children and classes	School-based QRE* (*n*=319)	Demonstration protocol (*n*=319)	School observation	Number of child households for home study
1	Main Primary	Low land	Day	161 (5 classes)	32 grade 1 and 4 (2 classes)	32 grade 1 and 4 (2 classes)	1 week	4
2	Main primary	Low land	Tay, Kinh	89 (5 classes)	92 grade 1 and 4 (5 classes)	92 grade 1 and 4 (5 classes)	1 week	4
3	Satellite primary	Low land	Tay	256 (12 classes)	32 grade 1 and 4 (2 classes)	32 grade 1 and 4 (2 classes)	1 week	4
4	Satellite primary	High land	Xa Pho	67 (5 classes)	20 grade 1 and 4 (2 classes)	20 grade 1 and 4 (2 classes)	1 week	4
5	Secondary	Low land	Kinh, Tay, Day, Xa pho, Dao	247 (11 classes)	66 grade 7 (3 classes)	66 grade 7 (3 classes)	1 week	2
6	Secondary	Low land	Kinh, Tay, Day, Xa pho	328 (11 classes)	77 grade 7 (3 classes)	77 grade 7 (3 classes)	1 week	2

* QRE=questionnaire.

## Methods

In this study, both quantitative and qualitative methods were applied (Table 1). Quantitative methods included a school survey using a structured questionnaire and a demonstration protocol with first, fourth, and seventh graders. The selected children were assumed to represent different levels of exposure to school-based hygiene promotional activities (through lecture within national curriculum and extracurricular activities every month) and level of the Kinh fluency. The structured questionnaire, developed by the first author, was piloted among a group of 10 children and then revised before use. The field component of school survey was carried out over a 6-week period, from September to mid-October 2008. In total, 319 children who came from first, fourth, and seventh grades responded to the questionnaire during school time, with a 100% response rate (Table 2). The questionnaire was answered individually by each child in a separate and private location within the school premises. The questionnaire included questions about basic demographics (Table 2) and the HWWS behavior (Table 3). The questions concerning HWWS compliance included 1) ‘When did you wash the hand yesterday?’ and 2) ‘How did you wash the hand yesterday?’. Both these questions referred to the previous school day. HWWS reported by schoolchildren using water and soap during the behavior in the past 24 hours was defined as self-reported HWWS (Tables 3 and 6). Similarly, handwash (HW) at critical times was defined as HW before cooking, before eating, and after defecation (Table 3).

**Table 2:** Main characteristic of 319 schoolchildren participating structural questionnaire and demonstration in the study

Variable	Frequency	Percent
School type		
Secondary school	143	45
Primary school	124	39
Satellites	52	16
Grade		
1	62	19
4	114	36
7	143	45
Sex		
Male	165	52
Female	154	48
Ethnicity		
Kinh	7	2
Tay	163	51
Xa Pho	33	10
Day	116	36
Number of family members	319	5.5±1.5
Household own latrine	174	54
Household assets		
Having TV	246	77
Having radio	94	29
Having bicycle	177	55
Having motor-bicycle	169	53

**Table 3:** Handwashing practice of 319 rural school children from face-to-face interview, Vietnam, 2009

Variable	By gender			By grade			By ethnicity*		
		
	Total	Male	Female	Grade 1	Grade 4	Grade 7	Kinh-Tay	Xa Pho	Day
Sample size	319	165	154	62	114	143	170	33	116
When my hand is visibly dirty	23%	25%	21%	19%	19%	27%	25%	12%	23%
Before eating	60%	59%	60%	24%	57%	77%	63%	33%	62%
After defecation	21%	19%	23%	2%	24%	27%	24%	6%	22%
Before cooking	3%	4%	3%	0%	0%	8%	3%	0%	5%
After playing in yard	8%	9%	8%	3%	9%	10%	7%	15%	9%
Other times	41%	38%	45%	34%	34%	50%	39%	39%	44%
HW at critical times	1%	1%	2%	0%	0%	3%	1%	0%	2%
Water alone	31%	30%	31%	40%	26%	30%	26%	52%	32%
Water and soap	66%	67%	66%	32%	68%	80%	71%	39%	66%
Other materials	4%	6%	3%	3%	3%	6%	4%	0%	7%

Shading denotes significant results.

* Kinh and Tay was merged into one group because the number of Kinh student was very small (7).

To explore the skill of HWWS of schoolchildren, a demonstration protocol was developed based on eight steps of HWWS, guided by the Ministry of Education and Training, including 1) dip hands into water, 2) wash the hand with water, 3) use soap on the hands, 4) wash the hollow of the hands at least three times, 5) wash the back of the right hand at least three times, 6) wash the back of the left hand at least three times, 7) wave the hands, and 8) dry the hands with a towel (28). A schoolchild after answering the questionnaire would be asked to practice the behavior using the materials suggested (e.g. water, soap, and towel). Steps involved in HWWS were noted, and the time taken for HWWS was recorded using a clock.

The qualitative method used spot observation in six schools and in the homes of 24 schoolchildren to learn about the physical environment for HWWS behavior at school and at home. The school selection was based on two main criteria: 1) the school had students of all grades (for primary level, from grade 1 to 5; secondary level, from grade 6 to 9) and 2) logistic issue. Structured and participative observations were carried out for a week at each school. The observation also included data collection using a checklist on handwashing before eating at school (such as snack).

For home observation, 20 homes comprising 24 children (8 in highland and 12 in lowland) in four villages were selected and observed for 76 days, with 3–5 days for one home observation (29). The selected homes were purposively from those schoolchildren participating in the structural questionnaire at school. The households had one or more children studying either in grade 1 (six children), grade 4 (nine children), or grade 7 (nine children) at one of the six selected schools, including 10 males and females. In addition, the household selection represented four different ethnic groups (Kinh, Xa Phó, Tày, and Dáy) in the two communities. Each schoolchild was observed for 3–5 days, from the beginning of a school day at 7 AM and followed during the whole day until evening at their homes, that is, 8 PM. We observed the physical environment (water, soap, towel …); HWWS education; school support from teachers; support from peers, parents, and others toward promoting the practice of HWWS among schoolchildren, both at school and at home.

All face-to-face interviews and observation at home and school were carried out by a research team that composed of the first author and three research assistances. All interviews were conducted in Vietnamese, specifically the Kinh language, except in the case of 12 schoolchildren of grade 1 from a highland community who were supported by a local translator.

### Data analysis

All quantitative data were checked and quality assured and entered into EPI-INFO by the research team. The author analyzed the data using STATA (30). A descriptive analysis of HWWS compliance (when, how, how long) by schoolchildren was performed (Tables 3, 4, and 5). Eight steps of HWWS guided by the Ministry of Education and Training were used for the analysis (Table 4) (28).

**Table 4:** Demonstration results of Handwashing practices of 319 rural school children, Vietnam, 2009

Variable	By gender			By grade			By ethnicity		
		
	Total	Male	Female	Grade 1	Grade 4	Grade 7	Kinh-Tay	Xa Pho	Day
Sample size	319	165	154	62	114	143	170	33	116
Dip hands into water	82%	81%	83%	74%	78%	88%	84%	67%	84%
Wash the hand with water	46%	48%	44%	55%	51%	38%	44%	46%	48%
Use soap on the hands	78%	79%	77%	60%	75%	89%	82%	67%	76%
Wash the hollow of hands at least 3 times	69%	73%	64%	68%	61%	76%	75%	39%	67%
Wash the back of right hand at least 3 times	52%	60%	44%	39%	46%	64%	60%	24%	49%
Wash the back of left hand at least 3 times	49%	55%	42%	34%	41%	61%	54%	24%	48%
Wave hands	64%	66%	62%	58%	67%	64%	67%	55%	63%
Dry hands with towels	46%	39%	53%	21%	44%	58%	55%	15%	41%
Proper HWWS*	3%	4%	2%	2%	4%	4%	5%	0%	2%

Shading denotes significant results.

* Denote for the schoolchild practice HWWS following 8 steps guided by Ministry of Education and Training.

**Table 5:** Distribution of time for HWWS practices of 319 rural school children, from demonstration, Vietnam, 2009

Variable	By gender			By grade			By ethnicity		
		
	Total	Male	Female	Grade 1	Grade 4	Grade 7	Kinh-Tay	Xa Pho	Day
Sample size	319	165	154	62	114	143	170	33	116
>60 sec	17%	22%	11%	29%	15%	13%	15%	24%	18%
30–60 sec	58%	57%	60%	34%	61%	67%	62%	39%	58%
<30 sec	25%	21%	29%	37%	25%	20%	23%	36%	24%
Mean Standard deviation	45.3	49.2	41.2	49.1	44.5	44.4	45.7	40.3	46.2
	26.63	28.76	23.54	34.38	26.72	22.47	23.79	24.92	30.79

Shading denotes significant results.

Multivariate logistic regression modeling was performed to examine the association of the self-reported HWWS behavior with relevant demographic factors (Table 6). The dependent variable was the self-reported HWWS (yes/no). The independent variables included grade, school type, gender, ethnicity group, owning a home latrine, and household assets. ‘Cluster’ option was introduced in the model to control for the dependence between students from the same class and school. Odds ratios with 95% confidence intervals were used. Chi-square test, *t*-test, and a significance level at 0.05 were used.

**Table 6:** Association between self-reported HWWS with selected demographic factors among 319 rural schoolchildren, Vietnam, 2009

Variable	OR	95% CI
Primary school (vs. secondary school)	1.13	0.55–2.29
Grade 4 (vs. grade 1)	4.14	2.00–8.56
Grade 7 (vs. grade 1)	7.76	3.67–16.40
Male (vs. female)	0.97	0.57–1.62
Tay group (vs. Kinh-Tay)	0.79	0.44–1.44
Day group (vs. Kinh-Tay)	0.28	0.11–0.70
Household own latrine (yes vs. no)	0.69	0.40–1.22
Having TV (yes vs. no)	0.70	0.35–1.38
Having radio (yes vs. no)	1.17	0.66–2.07
Having bicycle (yes vs. no)	0.89	0.50–1.58
Having motor-bicycle (yes vs. no)	1.39	0.77–2.50

Shading denotes significant results.

All observation notes were recorded and analyzed using a thematic approach. The theme included 1) physical environment for HWWS (availability of soap and water) and 2) school and home activities enable schoolchildren to practice HWWS.

This study was approved by the Ministry of Health; the Ethical Committee of National Institute of Hygiene and Epidemiology; and the authorities at the school, community, and village levels. In this article, names of all schools, villages, and communities are concealed, and schools are referred to by the area they belong to (main or branches) to ensure the anonymity of informants.

## Results

### HWWS compliance

In this study, individual interviews demonstrated that schoolchildren did not commonly wash their hands, even with water (Table 3). The common time for HW was before eating (60%). Only 23% of schoolchildren reported HW after defecation. Very few did before cooking (only 7 students out of 319; generated 2% reported). Only four students (three female and one male student at the secondary school level) reported to HW at all critical times (before cooking, after defecation, and before eating).

Table 3 also demonstrated that 66% of respondents did HW with soap the previous day. HW is similar for both male and female students but significantly different with regard to grade and ethnicity; higher the grade, the higher the tendency to HW (32% among grade 1 and 80% among grade 7), with *p*<0.05. Likewise, the students belonging to the Xa Phó group did not practice this much (39%), compared to the other groups (Kinh-Tay, 71% and Day, 66%), *p*<0.05.

Observation both at school and home revealed that almost all students did clean the playground with their hands for playing certain games (exposure to dirt/soil) but did not HW after these activities. School observation results show that about half of more than 1,000 students observed at school snacked before school hours (probably breakfast) and during break time (from a vendor near the school) without HW before eating. Furthermore, some students did HW but with unclean water (visibly seen) from drains and water holes in the school playground.

Through demonstration protocol, children demonstrated low level of HWWS skills. Table 4 shows that the percentage of students who demonstrated HWWS was 78%. However, out of 319 participants, only 10 schoolchildren could do HWWS correctly, as per the guidelines of the Ministry of Education and Training (28). The actions less frequently done by children included: drying hands with towels (46%), washing the backs of both hands, and rinsing hands with water before using soap (Table 4).

Table 5 shows that the average HWWS time was 45 sec (±26.63). The HWWS time significantly differed by gender. The percentage of students washing their hands in the recommended time (30–60 sec) was 58%, and it increased by grade (from 34% among grade 1 to 67% among grade 7; *p*<0.05). Interestingly, male students washed their hands longer than the female students (49 sec vs. 41 sec; *p*<0.05).

Table 6 presents the logistic model analysis between the self-reported HWWS rate and relevant demographic factors. Grade and EMG were associated with the behavior significantly. Older children reported more frequent HWWS [grade 4 vs. grade 1: OR=4.14 (2.00–8.56), grade 7 vs. grade 1: OR=7.76 (3.67–16.4)], and the behavior was less common among the students belonging to the Xa Phó group [Xa Phó vs. Kinh-Tay: OR=0.28 (0.11–0.70)].

### The physical environment for HWWS at both school and home settings

Observation at school shows that out of six schools, HWWS station was absent or not functional at four schools. In two schools with HWWS station installed outside the latrines, only one had water supply. All six schools did not have soap for HW. All six schools had no specific lesson on HWWS for schoolchildren or guidelines for them to practice during the 6-week observation at school, as stipulated in the national routine school program or curriculum.

Observation at home found that children had good opportunity to access water and soap for HW. All 20 households of schoolchildren had soap and water available for HW. Eight households had latrine; however, the location of the latrine was not close by. In addition, schoolchildren were not reminded to HW with soap during observation time.

## Discussion

This study demonstrated the low rate of HWWS among schoolchildren and the poor physical environment at school and home that inhibited them from practicing the behavior.

The study found that the schoolchildren did HWWS more commonly before eating but very few did so after defecation and before cooking. The HWWS rate was similar for boys and girls. A poor rate of HWWS was in line with what other studies previously had shown in rural Senegal (21) and Ethiopia (19). In this study, the HWWS increased significantly by grade, with the lowest at grade 1 (32%) to the highest at grade 7 (80%). The significant difference in HWWS with regard to grade or age group of schoolchildren can be explained with the fact that the students in higher graders have greater exposure to public and school hygiene promotion, as found in rural Cambodia (20). The finding underlines the importance of targeting the younger schoolchildren in HWWS education at school.

The study also found that ethnicity also had an impact on self-reported HWWS, with this behavior less common among highlanders or Xa Phó (Tables 3 and 6). In fact, in the highland communities where the Xa Phó ethnic group lives, the parents of the schoolchildren often go far away from home for farming activities, work long hours, and have limited opportunities to remind their children about hygiene practices (31, 32). Thus, not only ethnicity but also economic conditions have a negative impact on the HWWS compliance of schoolchildren in the multiethnic population.

The study also found that physical environment at school was poor, while all homes visited had water and soap. Similarly, availability of water and soap had a great influence on HWWS compliance of schoolchildren, as found in previous studies (22, 26). In addition, the study also found that HWWS was not a part of school and home schedule, where nobody reminded schoolchildren to HWWS, such as requesting them to HWWS before eating or cooking. It might reflect failure of adults (teachers and parents) to reinforce HW behavior. The findings implied that there was a need to prioritize HWWS among schoolchildren in the multiethnic population to reduce the high-burden of hygiene-related diseases among them. An intervention can be made in school settings, through a provision of soap and water facilities at a low-cost, with new HWWS lessons placed in the curriculum for grade 1. In addition, there should be motivation for schoolchildren to HWWS at home, where soap and water are available, by reminding them ‘clean hand to be a good child’ or through peer-to-peer education.

Some authors argued that studies on hygiene using only questionnaire-based surveys often have limited value (5, 33). However, others found that hygiene studies of smaller schoolchildren can be done with questionnaires because students express their practice truthfully and not provide desired answers or answers influenced by adults (34). Children are often a more reliable source of information than adults (35) as they are not aware of what is socially desirable (21). Furthermore, some authors have argued that hygienic behaviour could be less varied at a population level than at an individual level (36, 37). Therefore, this study applied triangulation to describe the HWWS compliance of schoolchildren, including face-to-face interviews, demonstration protocol, and observation. The results from these methods could complement each other, for example, using questionnaire to describe HWWS behavior, demonstration for exploring HWWS skills, and observation of physical environment and support at school and at home. The consistency of the results of HWWS before eating between Questionnaire and Observation recommend using the questionnaire to explore the hygiene behaviours of schoolchildren, particularly for 4th graders (Questionnaire: 57%; Observation: 50%). However, the HWWS of two key players, teachers and parents, might have a great influence in shaping the hygiene behavior of schoolchildren, and it should be further studied.

In sum, the study demonstrated the poor level of HWWS among schoolchildren in the multiethnic population of rural Vietnam, which needs more attention from policy makers. Education on handwashing needs to be prioritized to multiethnic children at school.
